# Outcomes of Resident-versus attending-performed Tube Shunt Surgeries in a United States Residency Program

**DOI:** 10.5005/jp-journals-10008-1245

**Published:** 2018-08-01

**Authors:** Loka Thangamathesvaran, Elliot Crane, Kunjal Modi, Albert S Khouri

**Affiliations:** 1 Medical Student, Department of Institute of Ophthalmology and Visual Science, Rutgers-New Jersey Medical School, Monmouth Junction, New Jersey, USA; 2 Ophthalmology Resident, Department of Ophthamology, New Jersey Medical School, Newark, New Jersey, USA; 3 Glaucoma Fellow, Department of Ophthamology, New Jersey Medical School, Newark, New Jersey, USA; 4 Associate Professor, Director of Glaucoma Division, Residency Program Director, Department of Ophthamology, New Jersey Medical School, Newark, New Jersey, USA

**Keywords:** Cohort study, Educational training, Glaucoma, Resident versus attending, Tube shunt surgery

## Abstract

**Introduction:**

Glaucoma is a chronic optic neuropathy with increasing global prevalence, necessitating trainees in ophthalmology to be well-trained in the surgical modalities used to manage glaucoma. It is also important to not compromise patient safety and treatment efficacy for training and education. The purpose of our analysis is to compare postoperative outcomes of resident versus (vs.) attending performed tube shunt surgeries (TS).

**Materials and methods:**

A retrospective, chart review was performed of patients who had undergone TS between 2009 and 2015 at Rutgers University in Newark, New Jersey, USA. Inclusion criteria was patients with a confirmed diagnosis of glaucoma, who underwent either an Ahmed or Baerveldt TS, and had at least two evaluation visits before the surgery to establish baseline characteristics. Exclusion criteria were patients with follow up for less than 1 year. The main outcome measure was the surgical success at 1 year follow up after TS. Surgical success was defined according to recommendations from the Glaucoma Surgical Trials guidelines published by the World Glaucoma Association (WGA):

• 20% reduction in IOP and absolute IOP ≤ 21 mm Hg (criteria 1)

• 30% reduction in IOP and absolute IOP ≤ 18 mm Hg (criteria 2)

**Results:**

A total of 120 cases: 60 attending and 60 resident cases that met all the inclusion criteria and none of the exclusion criteria were included. The mean intraocular pressure (IOP) one year post surgery were 15.06 ± 3.55 and 15.21 ± 5.17 mm Hg for attendings and residents respectively (p = 0.422). At the 1 year time point, 87% of resident cases and 95% of attending cases met the qualifications of criteria 1 for success. Kaplan Meier analysis was performed and did not show a significant difference in the outcome (p = 0.325). At the 1 year time point, 80% of attending and resident cases met the qualifications of criteria 2 for success. Kaplan-Meier analysis was performed and did not show a significant difference in the outcome (p = 0.401). There were no differences in complication and failure rates between resident and attending performed cases. Resident-performed cases had a longer intraoperative time in comparison to attending performed cases (p = 0.02).

**Conclusion:**

Resident-performed surgeries are as effective as attending performed surgeries. Resident-performed TS does not compromise safety and better prepares future physicians to deliver optimal care.

**Clinical Significance:**

Attendings may consider incorporating more resident performed, attending supervised TS procedures into their clinical practice as surgical training to manage common ophthalmological conditions like glaucoma is essential to residency training.

**How to cite this article:** Thangamathesvaran L, Crane E, Modi K, Khouri AS. Outcomes of Resident-versus attending-performed Tube Shunt Surgeries in a United States Residency Program. J Curr Glaucoma Pract 2018;12(2):53-58.

## INTRODUCTION

Glaucoma is a chronic optic neuropathy caused by elevated IOP that leads to the progressive loss of peripheral and central vision. The global prevalence of glaucoma is expected to increase with some estimates approximating 111.8 million people living with glaucoma by 2040.^1^ These astounding figures illustrate the need for future ophthalmologists to have proper training in managing the condition. Common surgical modalities to manage this condition include trabeculectomy and TS. For a long time, trabeculectomy was the gold standard surgical method, but with evidence of similar efficacy and safety, management preferences have changed favoring the use of TS to manage glaucoma.^2,3^ The American College of Graduate Medical Education recommends residents complete at least five filtering or TS as primary surgeons before graduation.^4^

Surgical training demands practice to attain competency, but efficacy and patient safety cannot be compromised in the training period. Previous studies have examined the safety of these procedures when performed by residents.^5^ Although residents had successful outcomes and lower complication rates after performing both trabeculectomy and TS, better results were observed with TS. However, no study that we are aware of has thus far been conducted directly comparing resident and attending tube surgery outcomes and complications. A similar analysis between resident and attending performed cases has been done for trabeculectomies with results showing comparative outcomes and complication rates.^5^

## MATERIALS AND METHODS

This study was approved by the Institutional Review Board at Rutgers University Hospital, Newark, New Jersey, USA and was ethically conducted in accordance with the declaration of Helsinki. A retrospective chart review was conducted to identify patients who underwent TS for glaucoma between January 2009 to December 2015 at Rutgers University Hospital. In total, 270 cases were identified, and 153 cases were chart reviewed. Inclusion criteria were patients with a confirmed diagnosis of glaucoma, who underwent either an Ahmed or Baerveldt TS, and had at least two evaluation visits before surgery to establish baseline characteristics. A diagnosis of glaucoma was confirmed through the use of several modalities including the clinical exam, and structural and functional tests like the optical coherence tomography (OCT) and visual field testing. The mean intraocular pressure from two previous visits were used as a baseline to account for day to day fluctuation. Exclusion criteria were patients with follow up for less than 1 year. Attending and resident cases were incorporated into the study after matching for several preoperative variables. They included subject age, glaucoma subtype, preoperative intraocular pressure, number of preoperative glaucoma medications, and preoperative visual acuity (Table 1). The intraocular pressures were matched within a ± 5 mm Hg range and the number of glaucoma medications were matched within ± 1 medications. In total, 60 resident and 60 attending cases were identified and incorporated into the study.

Preoperative data collected included: age, sex, baseline applanation intraocular pressure, glaucoma subtype, ocular history, visual fields (VF), lens status, visual acuity, number of glaucoma medications, and cup to disc ratios. Visual acuity was converted to log MAR values as follows: counting fingers, 2.0; hand movements, 2.5; perception of light, 3.0; no perception of light, 3.5.

Intraoperative data collected included tube shunt type, tube location, operative time, and intraoperative complications. Post-operative data collected included visual acuity, applanation intraocular pressure, and number of glaucoma medications at the 1 month ± 2 weeks, 3 months ± 1 month, 6 months ± 2 months, and 12 months ± 2 months time points. The following postoperative complications were noted: corneal edema, tube removal, tube revision, exposure, hyphema, conjunctival dehiscence, corneal haze, and endophthalmitis.

Success criteria was defined as the following and adopted from the guidelines on design and reporting of Glaucoma Surgical Trials published by the World Glaucoma Association (WGA).^6^

**Table 1:** **Table 1:** Patient pre-operative demographic comparison between the resident and attending cases

		*Attending*		*Resident*			
*Number of eyes:*		60		60			
*Laterality:*							
Left		31		31			
Right		29		29			
*Gender:*							
Male		28		34			
Female		32		25			
*Average age:*						p-value	
Mean ± SD		63.91 ± 18.32		58.11 ± 16.35		0.072	
*Medical comorbidities:*							
Hypertension		13		13			
Diabetes		4		5			
Both		17		12			
None		26		30			
*Glaucoma subtype:*						X2	
Uveitic		15		17		0.67	
POAG		29		25		0.44	
CACG		4		7			
Traumatic		5		3			
Neovascular		5		5			
Exfoliation		1		0			
Steroid induced		0		1			
Congenital		1		2			
*Pre-operative cup:disc ratio*						p-value	
Mean ± SD		0.84 ± 0.16		0.85 ± 0.16		0.482	
*Pre-operative lens status:*							
Phakic		21		33		X2	
Pseudophakic		39		24		0.03	
Aphakic		0		3		0.01	
*Previous glaucoma laser:*							
Laser		4		5			
trabeculoplasty							
Peripheral		16		18			
iridotomy							
Cyclophotoco-agulation		0		6			
*Visual fields^a^:*						p-value	
Mean defect (MD)							
Mean ± SD		–15.23 ± 10.04		–20.68 ± 10.27		0.052	
Pattern standard							
deviation (PSD)							
Mean ± SD		7.30 ± 3.83		6.42 ± 3.50		0.213	
*Intraocular pressure (mm Hg)*						p-value	
Mean ± SD		30.19 ± 9.00		30.37 ± 7.96		0.45	
*Number of glaucoma medications taking prior to surgery*						p-value	
Mean ± SD		3.08 ± 1.16		3.48 ± 1.38		0.09	
*Visual acuity:*						p-value	
Log MAR							
Mean ± SD		0.87 ± 0.82		1.23 ± 0.93		0.21	
Snellan conversion		20/148		20/340			

^a^values as defined by FL < 30%, FN/FP < 30%*

- 20% reduction in IOP and absolute IOP ≤ 21 mm Hg (criteria 1)
- 30% reduction in IOP and absolute IOP ≤ 18 mm Hg (criteria 2)

**Table 2:** **Table 2:** Patient intraoperative comparison between resident and attending cases

		*Attending*		*Resident*		*p-value*	
*Tube Implanted:*							
Ahmed		34		52			
Baerveldt		24		7			
Molteno		1		1			
Not specified		1		0			
*Tube placement:*							
Superotemporal		57		58			
Superonasal		1		2			
Inferonasal		2		0	
Intraoperative time (first incision to closure in minutes)		50 ± 9.07		55 ± 15.95		0.02	

*Statistically significant difference from baseline

**Fig.1:**
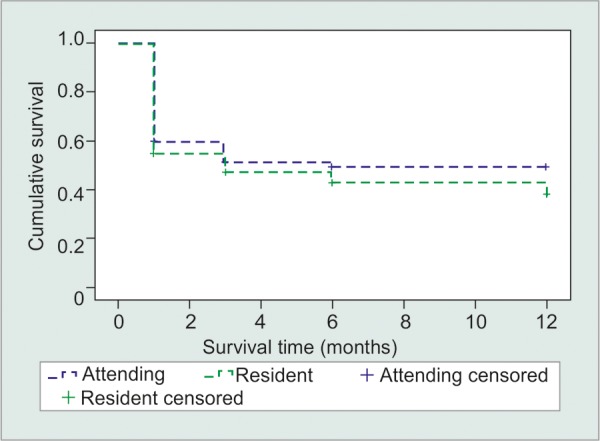
Kaplan-Meier curve for criteria 1: 20% reduction in IOP and absolute IOP ≤ 21 mm Hg (p = 0.325)

Failures were also defined according to WGA guidelines as no light perception, a second glaucoma surgery done to control intraocular pressures, and persistent hypotony defined as < 6 mm Hg on two consecutive visits after 3 months post-surgery.

Statistical analysis was carried out using two tailed t-test, chi-squared test, fisher’s exact test, and Kaplan-Meier. Statistical package for the social sciences (SPSS) version 22 (IBM, Armonk, New York, USA) and Excel (Microsoft, Redmond, Washington, USA) were used to perform this analysis. A p-value of < 0.05 was considered significant.

## RESULTS

A total of 120 cases-60 resident and 60 attending performed TS fit the above inclusion and exclusion criteria and were included in this analysis.

Intraoperative characteristics are noted in Table 2. Resident-performed cases on average had longer operating times at 55 minutes compared to 50 minutes for attending performed cases (p = 0.02).

Postoperative characteristics at the 1 month, 3 months, 6 months and 12 month time periods are listed in Table 3. The mean logMAR visual acuity at the 1 year time point was 0.96 ± 0.86 and 1.06 ± 0.94 for attendings and residents, respectively (p = 0.291). The mean intraocular pressures at the 1 year time point was 15.06 ± 3.55 mm Hg and 15.21 ± 5.17 mm Hg for attendings and residents, respectively (p = 0.422). The mean number of glaucoma medications used at the 1-year time point was 1.53 ± 1.14 and 1.86 ± 1.30 for attendings and residents respectively (p = 0.093). The mean post-operative cup to disc ratio was 0.84 ± 0.17 and 0.84 ± 0.17 for attendings and residents, respectively (p = 0.479). The mean postoperative VF mean defect (MD) was 15.35 ± 10.57 and -17.26 ± 12.78 for attendings and residents, respectively (p = 0.34). The mean postoperative VF pattern standard deviation (PSD) was 6.93 ± 3.83 and 6.06 ± 3.41 for attending and resident respectively (p = 0.262). No significant differences were noted when comparing pre-operative VF MD, VF PSD, and cup to disc ratio between the resident and attending groups.

Significant differences were found in mean intraocular pressure and number of glaucoma medications only at the 3 month time point. At 3 months, residents had a higher IOP of 19.89 ± 8.88 mm Hg compared to 16.93 ± 6.98 mm Hg for the attendings (p = 0.022). Patients who had undergone TS by residents were also taking a higher number of glaucoma medications to control their intraocular pressure at this time point, 1.82±1.45 compared to attending cases 1.38 ± 1.14 (p = 0.040).

At the 1 year time point, the cumulative probability of survival was 33% for resident cases and 49% for attending cases qualifying for criteria 1 (Fig. 1). Kaplan-Meier analysis was performed and did not show a significant difference in the outcome (p = 0.325). The analysis was performed at the 1 month, 3 months, 6 months and 12 month time points. Patients who did not have office visits within the time intervals defined above were censored from the data pool. The overall success rate for the same criteria looking just at the 1-year time point was 87% for residents and 95% for attendings.

**Fig. 2:**
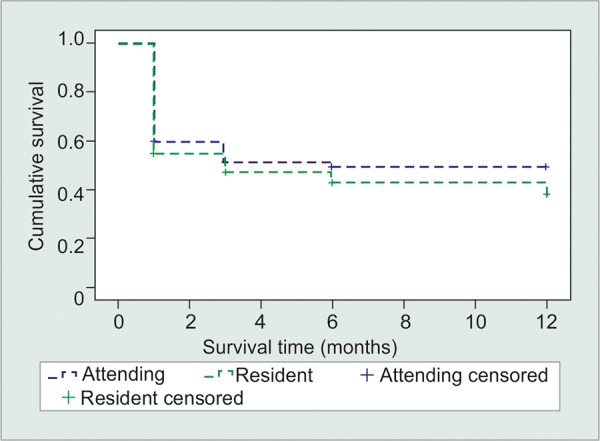
Kaplan-Meier curve for criteria 2: 30% reduction in IOP and absolute IOP ≤ 18 mm Hg (p = 0.401)

Similar analysis was performed for criteria 2. At the 1-year time point, the cumulative probability of survival was 28% for resident cases and 33% of attending cases (Fig. 2). Kaplan-Meier analysis was performed and did not show a significant difference in the outcome (p = 0.401). The success rate for the same criteria at the 1-year time point was 80% for residents and attendings.

The number of cases that met failure criteria are listed in Table 4. The most common failure in our analysis was a subsequent surgery to control intraocular pressure. Chi-squared tests were performed, and no statistically significant differences were found for any failure criteria. There were no intraoperative complications observed for any cases. Postoperative complications are listed in Table 5. Chi-squared tests were performed for each recorded complication, and no statistically significant differences were found. The three most common postoperative complications were- corneal edema, tube exposure, and hyphema.

**Table 3:** **Table 3:** Comparisons of attending and resident postoperative outcomes

		*Baseline*		*1 month*		*3 months*		*6 months*		*1 year*	
*Mean IOP (mm Hg):*											
Attending		30.19 ± 9.00		20.69 ± 10.23*		16.93 ± 6.38*		15.64 ± 6.04*		15.06 ± 3.55*	
Resident		30.37 ± 7.96		20.61 ± 10.46*		19.89 ± 8.88*		16.37 ± 5.98*		15.21 ± 5.17*	
p-value		0.45		0.32		0.02		0.31		0.422	
*Visual acuity (logMAR):*											
Attending		0.87 ± 0.82		1.08 ± 0.93		0.98 ± 0.92		1.06 ± 0.89		0.96 ± 0.86	
Resident		1.23 ± 0.93		1.29 ± 0.95		1.16 ± 0.95		1.17 ± 1.03		1.06 ± 0.94	
p value		0.21		0.12		0.17		0.29		0.29	
*Absolute pressure*				*Baseline to 1*		*Baseline to 3*		*Baseline to 6*		*Baseline to*	
*reduction:*				*month:*		*months:*		*months:*		*1 year:*	
Attending				9.28 ± 11.78		13.06 ± 9.23		14.14 ± 10.17		14.39 ± 9.79	
Resident				8.79 ± 10.36		11.31±11.74		14.65 ± 9.45		14.88 ± 8.76	
p-value				0.44		0.30		0.28		0.31	
*Relative pressure*				*Baseline to 1*		*Baseline to 3*		*Baseline to 6*		*Baseline to*	
*reduction:*				*month:*		*months:*		*months:*		*1 year:*	
Attending				0.27 ± 0.32		0.40 ± 0.23		0.44 ± 0.25		0.43 ± 0.21	
Resident				0.28 ± 0.36		0.32 ± 0.36		0.44 ± 0.24		0.46 ± 0.23	
p-value				0.46		0.08		0.50		0.23	
*# of glaucoma medications used to control IOP:*											
Attending		3.08 ± 1.16		0.98 ± 1.21*		1.38 ± 1.14*		1.39±1.25*		1.53 ± 1.14*	
Resident		3.45 ± 1.41		1.00 ± 1.33*		1.82 ± 1.45*		1.82±1.36*		1.86 ± 1.30*	
p-value		0.06		0.47		0.04		0.05		0.09	

*Statistically significant difference from baseline

**Table 4:** **Table 4:** Comparison of cases meeting failure criteria

		*Resident*		*Attending*		*Fisher’s exact*	
NLP		2		1		1.00	
Resurgery		10		5		0.27	
Hypotony		3		1		0.62	

**Table 5:** **Table 5:** Postoperative complication comparisons

*Complication*		*Attending*		*Resident*		*Fisher’s exact*	
Corneal edema		16		10		0.27	
Tube removal		1		1		1.00	
Tube revision		4		3		1.00	
exposure		10		10		1.00	
Hyphema		10		6		0.42	
Conjunctival		2		5		0.44	
dehiscence							
Corneal haze		9		4		0.24	
Endophthalmitis		1		2		1.00	

## DISCUSSION

To our knowledge, this is the first study to examine TS outcomes between residents and attendings. At the 1 year time point, there were no significant differences noted between resident and attending performed TS for any of the variables measured: IOP, number of glaucoma medications, complications, failures, relative intraocular pressure reduction, or absolute intraocular pressure reduction. These results demonstrate that quality and safety are not compromised in the educational training of the residents. Success rates for our study at the 1 year time point were 87% for residents and 95% for attendings as defined by criteria 1 and 80% for both residents and attendings as defined by criteria 2. These values mirror success rates from other studies, but a direct comparative analysis is difficult as most other studies primarily used IOP measurements as their sole success criteria, while in this study, IOP measurements and percent reduction were both used.^7^

Similar studies comparing the outcome of the resident versus attending performed surgeries have been published in multiple disciplines. A meta-analysis examining 182 studies by D’Souza et al. noted that although residents took significantly longer to complete the procedures, patient safety was not compromised.^8^ He examined in total 14 specialties, with 5% of publications in ophthalmology. Upon further subgroup analysis just limited to ophthalmology, no significant differences were found in the complication rate and duration of procedure between resident and attending performed cases (2 = 0.301, 2 = 0.164). However, the studies included in ophthalmology were limited to laser-assisted in situ keratomileusis (LASIK) surgeries, correction of entropion, pterygium surgery with autoconjunctival grafting, and cataract surgeries. Those analyses did not include glaucoma surgery.

Another meta-analysis examining 97 studies by Leeuw et al. reported increased operative times in resident performed cases, but no significant differences in outcome between resident and attending cases were noted.^9^ However, only one study comparing entropion correction outcomes was examined in ophthalmology with no subspecialty analysis performed, limiting the number of conclusions that can be drawn from this analysis.

The findings from our study corroborate the results from the ophthalmology studies included in the meta-analysis performed by D’Souza et al. demonstrating patient safety is not compromised in resident performed procedures. In our study, resident performed surgeries also had a longer intraoperative time. This time difference was small, 5 minutes, representing a 10% increase in operative time that would likely not affect patient outcome. This increased time can be attributed to multiple factors: time required for teaching, resident’s relative lack of experience, and time spent communicating and coordinating with another individual.

Our study and various other studies referenced above do not highlight a difference in patient safety and outcome between resident and attending performed procedures. However, as examined in one study by Asch et al., the educational training provided in residency does affect the future patient outcome. Obstetrics and gynecology training programs were rated in accordance with the number of maternal complications the associated hospital had.^10^ These rankings were then compared to the quality of care their graduates deliver. The hospitals that had more complications produced future physicians whose patients had higher complications. Another study also attributed better outcomes to physicians who were used to a higher volume of surgical procedures during their training.^11^ Although comparisons between specialties may be limited due to inherent differences between specialties, these findings emphasize the importance of high-quality surgical training during residency training to better prepare future physicians.

Simulations have an increasingly important role in current surgical training. Within the field of ophthalmology, virtual and wet field simulators are used as part of the resident curriculum to improve mastery of surgical technique and patient outcome, most specifically in cataract and vitreoretinal procedures.^12,13^ Although no specific training exists for TS, improved overall dexterity afforded by simulators can enhance the clinical results across a wide spectrum of procedures. In the specific context of glaucoma, technological integration has primarily been in the context of applications such as *Eye Handbook* that allow practitioners to calculate glaucoma risk tailored to each patient’s physical exam and history, and *Glaucoma* from will eye for patients to learn how to take a visual field exam, serve as a reminder to take eye drops, and keep track of intraocular pressure. Implementation of simulation training specific to glaucoma surgeries and management of its complications is still warranted. Furthermore, even within the existing simulation curricula, there is inadequate, objective appraisal of the intricacies within this teaching modality such as most effective training schedule and role of instructor in the educational process, thereby overall limiting the capacity to improve upon the shortcomings that exist in the system.

There were several limitations to our study and data analysis. First, our data does not specify the level of training or time of the year at which residents performed these cases. Although the majority were performed by senior residents in the last year of training (PGY4), some could have been performed by lower level residents (PGY2-3). There can be a difference in outcome and complication rate when comparing early third year versus late third year resident performed glaucoma surgeries.^14^ Furthermore, the complexity of each TS was not measured, and the possibility of an inherent bias with senior attending physicians completing more complex cases in comparison to resident physicians cannot be excluded, minimizing the difference in clinical outcome noted between the two cohorts. However, it is important to note teaching hospitals tend to shoulder more complex cases in comparison to private practices, potentially limiting this discrepancy.^15^ Next, the applicability of the conclusions drawn from this data is limited as this study covers the outcomes from a single institution. Finally, our examination only extended to the 1 year time point. A similar study done by Kwong et al. comparing resident versus attending outcomes following trabeculectomies found similar outcomes in the initial period, however, after the 24 month period there was a statistically significant decline in visual acuity of resident performed procedures that were not noted in attending performed procedures.^5^ The primary reason was noted to be the development of cataracts. In Kwong’s study, cases performed by attend-ings were more likely to undergo a subsequent cataract procedure following a trabeculectomy compared to resident cases, and this variation could have altered the outcomes in visual acuity. Our study extended for only one year, so changes beyond that time point are not included. It is also important to note that about 50% of phakic patients who undergo either trabeculectomy or TS will develop a cataract within 5 years.^16^ In our cohort of patients, 35% of attending cases and 55% of resident cases were phakic prior to the surgery (2 = 0.027), even though baseline VF and visual acuity were not different between resident and attending groups (p = 0.212 and p = 0.211 respectively). Since more attending cases had a preoperative cataract procedure, they had less potential to develop a cataract in the postoperative period.

## CONCLUSION

Overall, our study showed that in this cohort, resident performed surgeries were as effective as attending performed surgeries. Resident-performed TS does not compromise safety or efficacy, and better prepares future physicians to deliver optimal care.

## CLINICAL SIGNIFICANCE

Attendings may consider incorporating more resident performed, attending supervised TS procedures into their clinical practice as surgical training to manage common ophthalmological conditions like glaucoma is essential to residency training.
